# High miR-511-3p Expression as a Potential Predictor of a Poor Nutritional Status in Head and Neck Cancer Patients Subjected to Intensity-Modulated Radiation Therapy

**DOI:** 10.3390/jcm11030805

**Published:** 2022-02-02

**Authors:** Marcin Mazurek, Radosław Mlak, Iwona Homa-Mlak, Tomasz Powrózek, Anna Brzozowska, Wojciech Kwaśniewski, Grzegorz Opielak, Teresa Małecka-Massalska

**Affiliations:** 1 Department of Human Physiology, Medical University of Lublin, 20-080 Lublin, Poland; radoslawmlak@gmail.com (R.M.); iwona.homa.mlak@gmail.com (I.H.-M.); tomaszpowrozek@gmail.com (T.P.); grzegorz.opielak@umlub.pl (G.O.); teresamaleckamassalska@umlub.pl (T.M.-M.); 2 II Department of Radiotherapy, Center of Oncology of the Lublin Region St. John of Dukla, 20-090 Lublin, Poland; annabrzo@poczta.onet.pl; 3 Department of Oncological Gynaecology and Gynaecology, Medical University of Lublin, 20-081 Lublin, Poland; wojciech.kwasniewski@umlub.pl

**Keywords:** head and neck cancer, cancer cachexia, malnutrition, radiotherapy, IMRT, miR-511-3p

## Abstract

Nutritional deficiencies, including malnutrition and its irreversible type cachexia, are often observed in patients with head and neck cancer (HNC). Among the various factors contributing to the occurrence of these disorders, inflammation seems to be crucial. The potential regulatory properties of miR-511-3p, e.g., post-translational alteration of expression of genes with protein products that are involved in inflammation, may be related to nutritional deficiencies observed in HNC patients. Therefore, the aim of our study was to assess the correlation between pretreatment miR-511-3p expression and nutritional status in patients undergoing radiotherapy (RT) due to HNC. In our retrospective study, 60 consecutively admitted patients treated with intensity-modulated radiotherapy (IMRT) due to advanced HNC were enrolled. The analysis of miR-511-3p expression was performed using real-time PCR. Significantly higher expression of miR-511-3p was observed in well-nourished patients compared to patients with moderate or severe malnutrition (*p* = 0.0001). Pretreatment expression of miR-511-3p may be a useful biomarker of nutritional deficiencies in patients subjected to IMRT due to HNC.

## 1. Introduction

Head and neck cancers (HNC) are predominantly localized in the pharyngeal, nasopharyngeal, laryngeal and oral regions [1]. According to the epidemiological data, HNC is diagnosed in approximately 630,000 patients per year, and the most common histological type is squamous cell carcinoma, accounting for 90% of cases [1,2]. In recent years, the incidence of HNC was stable or even seems to have been declining; however, in the HPV-positive population, there has been a rise by two third [2]. HNC treatment includes surgical procedures, radiation therapy (RT), chemotherapy (CTH) and chemoradiotherapy (C-RT) as well as a combination of these methods. Selecting the optimal treatment scheme depends primarily on the stage of disease and the patient’s performance status (PS). Since HNC patients are usually diagnosed at advanced stages of disease, RT has become the treatment of choice in clinical practice. However, in order to reduce the adverse effects associated with traditional RT, modifications to RT (e.g., intensity-modulated radiotherapy (IMRT)) are gaining popularity among clinicians. IMRT allows exposing tumor tissue to higher doses of ionizing radiation while avoiding exposure for sensitive tissues of the head and neck area (brain stem, spinal cord, parotid glands, optic and nerve), thus contributing to a reduction in treatment time and increased safety [3,4,5]. Use of precisely calculated radiation doses reduces the risk of both early and late toxicity: oral mucositis (OM), dysphagia and xerostomia [6]. For instance, treatment using IMRT alone, compared to three-dimensional conformal radiation therapy (3DC-RT), results in a reduction in the percentage of HNC patients requiring enteral feeding (49% vs. 72%, respectively). Similar results were noted in HNC patients when chemo-IMRT and 3DC-RT were compared (63% vs. 82%, respectively) [7].

Malnutrition is a process that often accompanies cancer [1,2,3,4]. Malnutrition is a state resulting from the lack of intake or uptake of nutrients, leading to altered body composition and body cell mass, which leads to impaired physical and mental activity. The European Society of Clinical Nutrition and Metabolism (ESPEN) guidelines on definitions and terminology of clinical nutrition propose an etiology-based approach, distinguishing disease-related malnutrition (DRM) with inflammation, DRM without inflammation, and malnutrition without disease [8]. Malnutrition during RT or C-RT treatment can be observed in 44–88% of HNC patients [9]. On the other hand, cancer cachexia is noted in 20.2%–32.2% of treatment-naïve patients with HNC [10,11]. It should be noted that cancer cachexia can be considered as a DRM type with ongoing inflammation [12]. It is suspected that the main mechanism responsible for the development of cancer cachexia is more pronounced inflammation manifested by an increase in pro-inflammatory cytokines: TNFα, INF-γ, IL-1, IL-8, CRP and IL-6 [8,9,13,14]. Critical weight loss (CWL) in cancer is characterized by unintentional weight loss of ≥ 5% in 1 month or ≥10% in 6 months (before diagnosis-related assessment). However, the determination of CWL during therapy (RT or especially C-RT) has a greater clinical significance. CWL is observed in 57% of patients with HNC undergoing RT (according to criteria: >5% of weight loss during the time from start to the 8th week of RT or loss of >7.5% until the 12th week of RT) [15,16]. One of the characteristic changes seen in CWL is immunosuppression expressed by a lower number of invariant natural killer T cells (iNKT cells) and T cells [17]. Nutritional deficiencies observed in HNC contribute to unfavorable outcomes of treatment, toxicity, prolonged hospital stay, worse quality of life, and shorter overall survival (OS) [9]. Therefore, studies aiming at searching for predictive and prognostic factors useful for HNC patients with nutritional disorders are warranted [15,18]. Biomarkers, that have already been studied and indicated as related to atrophy and adipose tissue loss include zinc-α2-glycoprotein (ZAG), fibroblast grown factor 21 (FGF21), activin A (ActA), myostatin (MSTN) and growth/differentiation factor 15 (GDF15). Known biomarkers of inflammation accompanying cachexia are tumor necrosis factor-alpha (TNFα), interleukin 6 (IL-6), IL-1β, IL-8, C-reactive protein (CRP), albumin, and monocyte chemoattractant protein-1 (MCP-1) [19]. Products arising as a result of muscles and fat wasting were also proposed as potential biomarkers of cancer cachexia: glycerol, β-dystroglycan, hexosyl-ceramides (HCERs) and lactosyl-ceramides (LCERs) [19]. Moreover, altered blood serum or plasma expression of miR-203 (high), miR-21 (high), or miR-130a (low) was indicated as related to cancer cachexia [20]. However, none of the mentioned cachexia-related markers are used in clinical practice as their use has not been validated [20]. On the other hand, according to GLIM recommendations, CRP can be used as a supportive proxy measure of inflammation in the diagnosis of chronic malnutrition [21]. Nevertheless, the assessment of cancer cachexia biomarkers may facilitate the identification of patients at risk and indicate those requiring careful monitoring of nutritional status. This will allow for the timely implementation of appropriate nutritional and/or supportive treatment and thus prevent further development of this life-threatening condition [20].

Micro RNA (miRNA) consists of short (approximately 22 nucleotides) non-coding fragments of RNA [22]. It is estimated that over 60% of human genes are regulated by miRNA. Numerous studies have shown that miRNAs play a crucial role in the neoplastic process, including cell death regulation, proliferation, signaling and the formation of metastasis [23,24]. On the basis of changes in the level of miRNA expression, detection and differentiation of many pathological conditions, including the development of cancer, are possible [24]. Nowicka et al. describes miRNAs as molecules that are stable in different biological materials (serum, saliva, fresh frozen and formalin fixed and paraffin embedded), even when stored for many years or in RNA-degraded samples. Moreover, this shows that miRNAs can be analyzed repeatedly during and after the completion of therapy [24]. One of the most important advantages of miRNA as a biomarker (e.g., as a predictive or prognostic factor) is the ability to regulate from several dozen to even several hundred different genes. Thus, the assessment of even a single miRNA can indirectly reflect a series of related changes (in genes and possibly encoded proteins) involved in the development of complex processes, such as malnutrition [25]. Despite the fact that the profiles composed of many miRNAs seem to have a higher diagnostic value, in the literature, there are also numerous reports on the use of single miRNAs as markers of metastasis, treatment outcome predictors (chemosensitivity and radiosensitivity), and prognostic factors of patients with HNC [26,27]. Many authors have demonstrated a correlation between various miRNA and the occurrence of nutritional disorders (malnutrition, cachexia, sarcopenia and CWL) in the course of neoplastic disease [28,29,30]. MiRNAs may facilitate cancer cachexia via influence on skeletal muscle metabolism. It is known that miR-1296-3p, miR-3184-3p, miR-423-3p, miR-199a-3p, miR-345-5p, 423-5p, miR-532-5p and let-7d-3p regulate myogenesis, muscle metabolism and inflammation in cancer cachexia [31]. MiR-511 is located in the 5th intron of the gene encoding the macrophage mannose receptor (also known as *MRC1* or CD206). Transcription and cleavage of the miR-511 sequence result in pre-miR-511 formation, which is further transported to the cytoplasm, where it undergoes additional cleavage, leading to hairpin type miR-511-3p–miR-511-5p duplex formation. In contrast to miR-511-5p, which is further degraded, miR-511-3p is highly conserved in humans and becomes a bioactive mature strand. Moreover, a positive correlation between miR-511-3p and *MRC1* gene expression was found. It is known that the *MRC1* gene is expressed by dendritic cells (DCs) and macrophages. High levels of the product of the *MRC1* gene were noted in anti-inflammatory, wound healing, and tumor-associated macrophages [32]. Moreover, it has been demonstrated, that MRC1 expression is downregulated by INF-γ and upregulated by IL-4 and IL-13 [33]. It should be noted that the correlation between miR-511-3p and the occurrence of nutritional deficiencies in HNC patients subjected to RT has not been studied so far. Nevertheless, based on a correlation between the expression of miR-511-3p and the inflammation that has already been demonstrated by other authors, we assume that this miRNA may be associated with nutritional deficiencies [34,35,36]. MiR-511-3p plays an important role in inflammation by the regulation of peroxisome proliferator-activated receptor γ (PPARγ) expression and Toll-like receptor 4 (TLR4) regulation in human DCs. PPARγ regulates many DC functions: antigen presentation, migration, activation and pro-inflammatory cytokine production. It was demonstrated that low PPARγ expression results in the increased production of cytokine with a known pro-inflammatory influence: IL-6. Endotoxin, like lipopolysaccharide (LPS) in low concentrations, activates PPARγ in DCs, which causes decreased inflammatory cytokine production. Downregulation of miR-511-3p increases PPARγ expression and leads to suppression of LPS-mediated inflammation (inhibition of NF-κB pathway). On the other hand, overexpression of miR-511-3p causes decreased activation of PPARγ and increased production of pro-inflammatory cytokines [34,35,36]. Other research suggests that human miR-511 also has a role in TLR4 signaling, although the specific mechanism remains unknown [32]. Moreover, based on information available in miRNA databases (Target Scan 7.2, miRDBase), it can be predicted that studied miRNA could potentially participate in the regulation of a number of genes involved in metabolism (*IGF1R, IGF2,* and *IGF2BP1*) or inflammation (*TAB1, TAB3, LTBP1, TRAF1, IL6ST, IL7R, IL17RA, IL17RD, IL1R,* and *TLR4*).

Therefore, the primary aim of this study was to assess the relationship between pretreatment expression of the miR-511-3p and nutritional status in patients subjected to IMRT due to HNC. The secondary aim of this study was the evaluation of the studied miRNA as a prognostic factor.

## 2. Materials and Methods

### 2.1. Characteristics of the Study Group

In this observational (retrospective) study, 60 consecutively recruited patients in advanced stages of HNC, who were treated at the Department of Oncology of the Medical University of Lublin in the period from 2014 to 2017 and met inclusion and exclusion criteria were enrolled. Inclusion criteria: age over 18 years, any gender, histopathologically confirmed head and neck cancer, treatment with the use of IMRT alone or subsequently to surgery, with or without sequential and/or concomitant CTH, receiving the total radiation dose. Exclusion criteria were as follows: active infection, autoimmune disease, any previous cancer, any coexisting cancer and any type of previous anti-cancer treatment except surgery. This study was designed based on the STROBE guidelines. The Bioethical Commission of the Medical University of Lublin approved this project (KE-0254/232/2014). Prior to study, all patients signed the informed consent form.

### 2.2. Treatment and Patient Assessment

The patients were treated with the IMRT technique using the ONCOR linear accelerator (Siemens). A total of 54–70 Gy was used, with a daily dose of 2 Gy. For the group at advanced stages of cancer, 35 fractions of irradiation were applied on tumor and enlarged lymph nodes (total dose of 70 Gy). For post-surgical patients with volume risk, 33 fractions of irradiation were used (total dose of 66 Gy). Further, 60 and 54 Gy irradiation doses were used for the group with average and low risk. Elective lymph nodes were treated with a dose of 54 or 60 Gy. In some patients, cisplatin and 5-fluorouracil were used in addition to IMRT (1–4 cycles of chemotherapy).

Nutritional status was assessed—measuring weight, BMI, BIA, NRS-2002 and SGA—and blood sampling was performed, in which routine laboratory parameters and levels of the studied miRNA were measured, for each person on the same day (1 timepoint: from 24 to a maximum of 72 h before the start of RT). Moreover, in order to evaluate whether patients develop CWL, weight and BMI were measured on each RT week. In all patients, parenteral nutrition was used only during RT. It was used only when nutritional deficiencies were noted and if compensation of the intake of nutrients in any other way was not possible. In parenteral nutrition infusion emulsions (e.g., Multimel- Baxter Polska), all necessary ingredients such as vitamins, electrolytes, and microelements, selected individually depending on patient laboratory results, were included. In some patients, oral nutritional support (e.g., Nutridrink, Danone, France) was also used during RT.

The patient’s PS was assessed according to the Eastern Cooperative Oncology Group (ECOG) scale. The Related Health Problems and the International Statistical Classification of Disease were used to assess alcohol consumption. Nutritional risk for patients was also assessed using the NRS-2002 (Nutritional Risk Screening 2002) criteria. Moreover, in order to assess malnutrition risk, the nutritional risk index (NRI) was calculated by using the following formula: NRI = [1.519 * serum albumin (g/l)] + 41.7 * (current weight/usual weight). The patients were grouped into four groups according to NRI score: no risk (>100), mild risk (97.5–100), moderate risk (83.5–97.5), and severe risk (<83.5) of malnutrition [37]. The nutritional status of patients was assessed by the subjective global assessment (SGA) scale. According to the SGA scale, patients were grouped into three categories: well-nourished patients (SGA-A), moderate malnutrition (SGA-B) and severe malnutrition (SGA-C). A critical weight loss (CWL) was defined as a weight loss of >5% from the beginning of RT to week 4 or >6.25% to week 7 of therapy [16]. The SGA, NRS-2002, and CWL were outcome measures reflecting nutritional status. Moreover, the group of smokers was divided into: current and former smokers. A person who has never smoked or who has smoked <100 cigarettes in their lifetime was defined as a non-smoker, while a person who has smoked >100 cigarettes during their life or is still smoking was defined as a former smoker or a current smoker [38].

### 2.3. miRNA Expression Analysis

From each patient, prior to any anti-cancer treatment (excluding surgery), 5 mL of peripheral blood was collected into EDTA-containing tubes. The samples were centrifuged for 15 min at 1000× *g*; and within the next 30 min, obtained plasma was collected and immediately subjected to miRNA isolation. The column method was used for miRNA isolation from 200 µL of plasma samples with a dedicated kit according to the manufacturer’s protocol (miRNeasy Serum/Plasma Kit, Qiagen, Hilden, Germany). Purified miRNA samples were reverse transcribed into complementary DNA (cDNA) with a dedicated kit (TaqMan Advanced miRNA cDNA Synthesis Kit, Thermo Fisher Scientific, Waltham, MA, USA). The amplification reaction was performed on a StepOnePlus device (Applied Biosystems, Foster City, CA, USA) based on the manufacturer’s protocol. In real-time PCR, 96-well plates were used and the total reaction volume was 20 µL per well. Each well was filled with 10 µL of TaqMan Fast Advanced Master Mix (Thermo Fisher Scientific, USA) and supplemented with 1 µL of TaqMan Advanced miRNA Assay (20×) (Assay name: hsa-miR-511-3p, Assay ID: 478969, Thermo Fisher Scientific, USA), 4 µL of RNase-free water and 5 µL of diluted (1:10) cDNA template. The thermal cycling conditions consisted of enzyme activation at 95 °C, 40 cycles of denaturation at 95 °C for 1 s and annealing and elongation at 60 °C for 20 s. Each sample was analyzed in triplicate. The level of miRNA-511-3p expression was normalized to miR-26a-3p as a reference assay using 2^-ΔΔCt^ and 2^-ΔCt^ formulae.

### 2.4. Bioelectrical Impedance Analysis

Bioelectrical impedance analysis (BIA) was used to obtain body composition parameters. BIA analysis was conducted using the ImpediMed bioimpedance analysis SFB7 BioImp device (Pinkenba, QLD, Australia). Despite the various limitations of the method, validation studies confirm that BIS correlates highly with isotope dilution analysis, DXA and other reference methods [39,40,41]. Fat mass (FM) and fat-free mass (FFM) were calculated with the use of BIA, the fat-free mass index (FFMI) and the normalized fat-free mass index (nFFMI) using the following formulae: FFMI [kg/m^2^] = FFM [kg]/(height [m])^2^; nFFMI[kg/m^2^] = FFMI [kg/m^2^]+ 6.1*(1.8 − height [m]).

### 2.5. Statistical Analysis

MedCalc 15.8 software (MedCalc Software, Belgium) was used to perform a statistical analysis of the data. Results were considered statistically significant at *p* < 0.05. Due to the fact that continuous variables had a non-normal distribution (assessed by the D’Agostino–Pearson test), non-parametric tests were used where applicable. Spearman’s rank correlation was used for correlation assessment between the studied miRNA levels and the clinical, demographic and nutritional variables. The Mann–Whitney U test was used for comparisons of the expression of the studied miRNA depending on the demographic and clinical variables and nutritional status. The analysis of ROC curves was used to determine the cut-off points and to assess the diagnostic usefulness of the studied miRNA in differentiating patients with different nutritional statuses assessed using the SGA and NRS-2002 scales and depending on the occurrence of CWL. For the univariable assessment of the risk of malnutrition (SGA), nutritional risk (NRS-2002), CWL, depending on clinical, demographic and epigenetic features, and the odds ratio (OR) with a 95% confidence interval (95% CI) were calculated; however, for the multivariable analysis, logistic regression was used (results were adjusted to statistically significant variables from the univariable analysis). Univariable OS analysis was performed using the two-sided log-rank test (with the calculation of the hazard ratio HR and 95% CI) and visualized with the Kaplan–Meier curves. Multivariable OS analysis was performed using Cox logistic regression models (results were adjusted to statistically significant variables from the univariable analysis).

## 3. Results

### 3.1. Charasteristic of Study Group

A total of 60 consecutively enrolled patients with advanced HNC were included in this study. No patient refused to participate in this study. Men predominated in the study group (85%). The median age of patients was 63 years (range: 42–87 years). In all patients, histopathological examination confirmed the presence of III (26.7%) or IV (73.3%) stage HNC according to the 7th edition of TNM classification. In the case of 38.3% of patients, the HNC was located in the oropharyngeal, and in 55% in the larynx area. All patients underwent IMRT. Surgical treatment was performed for 76.7% of the patients followed by IMRT alone for 46.7% and by C-RT for 30%. C-RT or RT alone, without previous surgery, was used in 13.3% and 10% of patients, respectively. In 93.48% of patients, tumor resection was extended by lymphadenectomy. The interval time between surgery and nutritional assessment averaged 71 days. Nutritional support in the post-operational period included use of PEG in 3.34%, and feeding tube in 33.33% of patients. Nutritional support in the post-operational time was used in average for 16 days. Oral nutrition support during RT was used in 5% of patients. Parenteral nutrition during RT was used in 11.7% of patients. Study group characteristics are presented in Table 1.

### 3.2. Nutritional Assasement

In our study, 46.34% of patients had a high risk of malnutrition (NRS > 3) according to the NRS 2002 scale. On the other hand, 48.3% of patients were moderately (B), and 36.7% were severely (C) malnourished according to SGA, whereas 33.2% of patients were diagnosed with CWL. According to the NRI, 88.33% of patients had moderate, and 5% had severe risk of malnutrition (Table 1).

### 3.3. Factors Affecting the Risk of Malnutrition According to the SGA Scale

In patients at the T4 tumor stage, the risk of moderate (B) and severe (C) malnutrition according to the SGA scale was nearly 27-fold higher (OR = 26.95; *p* = 0.0258). In patients abusing alcohol, a significantly higher risk (over 1.5 fold) of moderate and severe malnutrition (OR = 1.78; *p* = 0.0449) was found. In the case of smokers, the risk of moderate and severe malnutrition was over 4.5-fold higher (OR = 4.54; *p* = 0.0441). On the other hand, patients at the T4 tumor stage showed a significantly more than 4.5-fold higher risk of mild and moderate malnutrition (OR = 4.57; *p* = 0.0094) (Table S1).

### 3.4. Factors Affecting the Risk of Higher Nutritional Risk According to the NRS Scale

There was no statistically significant influence of any of the analyzed clinical and demographic factors on the occurrence of nutritional risk according to the NRS scale (Table S2).

### 3.5. Factors Affecting the Risk of CWL

Patients diagnosed with HNC in the oropharyngeal had an over 8-fold higher risk of CWL (OR = 8.04; *p* = 0.0007). On the other hand, the location of the tumor within the larynx was associated with an over 11-fold lower risk of CWL (OR = 0.09; *p* = 0.0004). There was a trend towards a significantly higher risk (approximately 3.8-fold) of developing CWL in patients with higher (≥3) nutritional risk according to the NRS scale (OR = 3.78; *p* = 0.0592) (Table S3).

### 3.6. miR-511-3p Expression in Predicting the Occurrence of Nutritional Deficiencies

**Figure 1:**
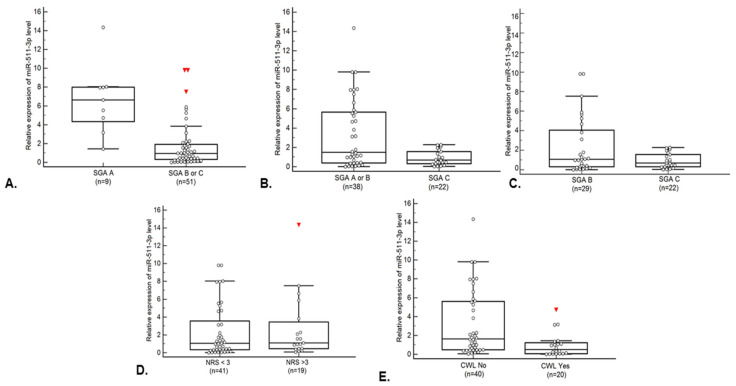
Relative expression of miR-511-3p level in group of patients: (**A**) well -nourished vs. moderately or severely malnourished (according to SGA); (**B**) well- nourished or moderately malnourished vs. severely malnourished (according to SGA); (**C**) moderately malnourished vs. severely malnourished (according to SGA); (**D**) with results <3 vs. ≥3 in scale of malnutrition risk (according to NRS-2002); (**E**) with vs. without critical weight loss. Abbreviations: CWL—critical weight loss, NRS-2002—Nutritional Risk Screening 2002, and SGA—subjective global assessment.

**Figure 2:**
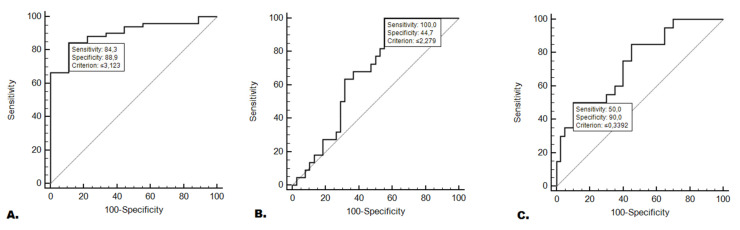
The assessment of the relative expression of miR-511-3p in predicting the occurrence of nutritional deficiencies was conducted using ROC analysis with AUC calculation. (**A**) Moderate or severe malnutrition (according to SGA); (**B**) severe malnutrition (according to SGA); (**C**) critical weight loss. Abbreviations: AUC—area under the curve, CWL—critical weight loss, SGA—subjective global assessment, and ROC—receiver operating characteristic.

Patients with moderate or severe malnutrition according to SGA showed significantly lower levels of miR-511-3p expression compared to well-nourished patients (0.93 vs. 6.27; *p* = 0.0001). Additionally, patients with severe malnutrition showed significantly lower expression of the studied miRNA compared to those that were well nourished or those that showed moderate malnutrition (1.49 vs. 0.68; *p* = 0.0251). Similarly, patients with CWL showed significantly lower levels of miR-511-3p compared to patients without CWL (1.64 vs. 0.51; *p* = 0.0025). The comparisons of the relative expression of miRNA-511-3p depending on the occurrence of specific nutritional deficiencies was presented in Figure 1A–E. Studied miRNA expression was 84.3% sensitive and 88.90% specific in predicting the occurrence of moderate or severe malnutrition (AUC = 0.90; *p* <0.0001; Figure 2A). In contrast, testing the level of miR-511-3p expression enabled the prediction of severe malnutrition with 100% sensitivity and 44.7% specificity (AUC = 0.67; *p* = 0.0110; Figure 2B). However, studied miRNA was characterized with insufficiently high diagnostic accuracy in differentiation of moderate and severe malnutrition according to SGA (AUC = 0.58; *p* = 0.3049). The analysis of this biomarker conducted to predict CWL was characterized by a sensitivity of 50% a and specificity of 90% (AUC = 0.74; *p* = 0.0003; Figure 2C). The lower level of miR-511-3p expression was associated with a significantly higher (over 37-fold) risk of moderate or severe malnutrition according to SGA (OR = 37.33; *p* = 0.0013). Moreover, the lower level of the tested miRNA was associated with a 17-fold higher risk of severe malnutrition (OR = 17; *p* = 0.0084). On the other hand, in patients with low expression of the studied miRNA, the risk of severe malnutrition (compared only with the moderate malnutrition) was significantly higher (more than 9 fold: OR = 9.45; *p* = 0.0411). In patients with low miR-511-3p expression, the risk of developing CWL was more than 7-fold higher (OR = 7.36; *p* = 0.0039). Detailed data including comparisons of miR-511-3p expression depending on nutritional status, evaluation of the diagnostic usefulness of miR-511-3p expression in predicting the occurrence of nutritional deficiencies and the assessment of the risk of nutritional deficiencies depending on the miR-511-3p expression is presented in Table 2.

### 3.7. Comparison of miR-511-3p Relative Expression Depending on Demographic, Clinical and Nutritional Variables

A significantly lower expression of miR-511-3p was observed in patients at the T4 tumor stage. Moreover, the IV stage of disease (according to TNM classification) was related to a lower level of the studied miRNA expression. Level of relative expression of the studied miRNA was not significantly related with nutritional support in the post-operational period and during RT (Table S4).

### 3.8. Correlation between miR-511-3p Expression and Nutritional Status Indicators

**Figure 3:**
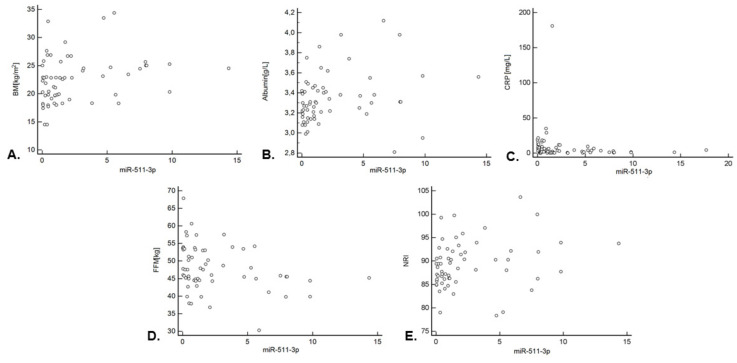
Correlation between the relative expression of miR-511-3p and nutritional status indicators: correlation between the relative expression of miR-511-3p and (**A**) BMI (kg/m^2^); (**B**) the level of albumin (g/L); (**C**) CRP [mg/L]; (**D**) FFM (kg); (**E**) the NRI. Detailed data are presented in Table 3. Abbreviations: BMI—body mass index, CRP—C-reactive protein, FFM—fat-free mass, and NRI—nutrition risk index.

A statistically significant weak, positive correlation was found between the BMI value and the level of miR-511-3p expression (rho = 0.317; *p* = 0.0136; Figure 3A). A statistically significant weak, positive correlation was also found between the concentration of albumin and the expression of the tested miRNA (rho = 0.322; *p* = 0.0094; Figure 3B). There was a weak, negative, significant correlation between CRP and miR-511-3p level (rho = −0.340; *p* = 0.0048; Figure 3C). Moreover, a weak, negative correlation was noted between the FFM and miR-511-3p expression (rho = −0.311; *p* = 0.0156; Figure 3D). A statistically significant weak, positive correlation was found between the NRI value and the level of miR-511-3p expression (rho = 0.317; *p* = 0.0130; Figure 3E). There was a weak, negative correlation of the FFMI (rho = −0.256; *p* = 0.0486) and a weak negative correlation of malnutrition according to the SGA scale (rho = −0.440; *p* = 0.0004) with miRNA expression level. There was a weak, negative correlation between the T tumor stage (rho = −0.296; *p* = 0.0218) and the studied molecule. A similar correlation was observed in the case of N feature (rho = −0.298; *p* = 0.0205). The stage of the disease was negatively, moderately correlated with the level of miR-511-3p expression (rho = −0.443; *p* = 0.0004) (Table 3).

### 3.9. Overall Survival

The univariable analysis showed that only patients with a poorer PS (>0) and with the M1 feature had a significantly higher (approximately 2- and 15-fold, respectively) risk of death (respectively: HR = 2.20; *p* = 0.0343; HR = 15.06; *p* = 0.0003).

The multivariable analysis (taking into account statistically significant variables from the univariable analysis) showed that only patients with the M1 feature had a significantly higher (approximately 11-fold) risk of death (HR = 10.81; *p* = 0.0473). The results of the analysis of the influence of demographic, clinical, nutritional and epigenetic variables on overall survival are presented in Table 4.

## 4. Discussion

Since there are no biomarkers specific to HNC-related nutritional deficiencies widely used in clinical practice (available scientific results are usually inconsistent, and promising factors have not been subjected to appropriate validation), studies of novel, potentially useful molecules are warranted.

It should be noted that the available literature lacks studies evaluating the relationship between miR-511-3p expression and nutritional deficiencies. In our study, we found that a lower level of miR-511-3p expression was associated with a higher risk of severe malnutrition and CWL in patients with HNC subjected to IMRT. Moreover, lower expression of the studied miRNA was found in patients at the T4 tumor stage. We also observed a weak correlation between miR-511-3p and BMI (positive), albumin (positive), FFMI (negative), malnutrition grade according to the SGA scale (negative) and the NRI (positive). According to our results, miR-511-3p might be related to both nutritional deficiencies (evaluated according to SGA and CWL criteria) and advancement of disease (T tumor stage). Thus, it is in accordance with the previously described versatility of miRNAs [28,29,30,31,32].

In our study, miRNA-511-3p had no influence on survival. On the other hand, we confirmed that known risk factors (PS, M feature) have a significant influence on the OS of patients treated by RT due to HNC.

Saroul et al. conducted a study on 90 patients with HNC in whom the nutritional, muscle function (Short Physical Performance Battery (SPPB)), and BIA parameters were assessed. Based on clinical data, the NRI, BMI, lean muscle mass, and the lean mass index were calculated. Additionally, the authors obtained a lumbar vertebral landmark (L3) muscle mass index (L3MMI). Patients with a low NRI score had significantly lower SPPB scores. Moreover, a significant correlation between the NRI and L3MMI was noted [42].

Lee et al. conducted a study on a group of 8-week-old C57BL/6L mice in whom Lewis lung carcinoma (LLC) cells (*n* = 8) or phosphate-buffered saline (PBS) (*n* = 6) was injected. The main aim of this study was to investigate the miRNA profile of skeletal muscle in a model of its atrophy caused by cancer cachexia. After 4 weeks of tumor development, the anterior muscles were used to obtain small RNAs and miRNA sequencing was performed. There was significantly higher expression of miR-511-3p in muscles of the study group compared to control (fold change: 1.53; *p* = 0.0024) [35].

In another study, the authors investigated the impact of miR-511-3p on PPARγ activity and DCs depended on regulation of LPS-induced inflammatory response. Before the examination of PPARγ expression, the human DCs were transfected with miR-511-3p inhibitors. In DCs with low expression of miR-511-3p, PPARγ expression was significantly higher (*p* < 0.01). In miR-511-3p-overexpressed cells, PPARγ expression was significantly decreased (*p* < 0.01) [36].

Labayen et al. studied whether the effect of birth weight on later body composition is modified by Pro12Ala polymorphism of the *PPARγ-2* gene. Authors conclude that in carriers of the Ala12 allele of the *PPARγ-2* gene, small body weight at birth may lead to lower FFM in adolescents [43].

It is believed that miRNAs may be involved in the development of inflammatory processes in muscle and adipose tissue [44]. Within the adipose tissue, the development of inflammation in the course of cachexia is associated with a change in the expression of miR-21, miR-155, miR-146a and miR-9, where they can influence the immune response through TLR [44]. It was demonstrated that the regulation of the NFκ-B pathway and the expression of TNFα is influenced by the level of miR-130a expression [45,46]. MiRNAs involved in the regulation of muscle mass in the course of cachexia include let-7d-3p, miR-345-5p, miR-423-5p and miR-486. Alterations in their expression contribute to the intensification of cellular processes that promote the development of muscle atrophy [19,47]. On the other hand, overexpression of miR-532-5p contributes to the development of cachexia related to the decrease in the secretion of neuropeptide Y (appetite regulation), while the reduction in the expression of genes regulated by miR-532-5p leads to the deactivation of mechanisms related to serotonin production (myogenesis) [47]. In the course of malnutrition, high expression of miR-486 leads to a decrease in muscle mass through activation of the insulin-like growth factor-1/ankyrin (IGF-1/Akt) signaling pathway [19,48]. Increased expression of miR-378 was observed in gastrointestinal cancer patients with cachexia. In this group of patients, miR-378 overexpression was associated with an intensified breakdown of adipose tissue. Increased lipolysis is stimulated, among others, by secreted catecholamines, which is manifested by an increase in characteristic proteins, i.e., perilipin-1 1 (PLIN1), lipase E (LIPE) or Patatin-Like Phospholipase Domain-Containing 2 (PNPLa2) [19,44,49]. High expression of miR-21 and miR29a was found to be involved in the induction of inflammation through activation of the gene (*TLR8*) encoding Toll-like 8 receptor [50]. On the other hand, in pancreatic and lung cancers, it was shown that miR-21 molecules secreted in the form of microbubbles bind to muscle cells, thus inducing their apoptosis. It was also proven that high expression of miR-206 and miR-21 reduce the activation of the eIF4E3 translation initiation factor and the YY1 transcription factor, which translates into the destruction of muscle cells, myogenesis arrest and the occurrence of muscle weakness [22,51].

In a study conducted by Powrózek et al. on a group of 70 HNC patients undergoing RT, the level of miR-130a expression and its impact on nutritional status were examined. It was found that the higher level of miR-130a expression was associated with a significantly higher risk of developing moderate and severe malnutrition according to the SGA scale (OR = 5.60; *p* = 0.039). On the other hand, patients with low levels of miRNA expression showed a 6-fold higher risk of weight loss >5% (OR = 6.34; *p* = 0.001), 14-fold higher risk of weight loss >10% (OR = 14.18; *p* < 0.0001) and a 3-fold higher risk of the significant decrease in BMI (OR = 3.41; *p* = 0.038) after the 7th week of RT. There was also a significant effect on the severity of cachexia depending on the level of miR-130a expression. Men with a weight loss >10% showed a significantly lower level of miR-130a expression compared to men with a weight loss >5% (*p* = 0.044). Patients with an elevated concertation of plasma TNFα and low expression of miR-130a had shorter OS compared to those with high TNFα levels and high miR-130a expression (HR = 2.84; 95% CI = 1.16–6.99; *p* = 0.029) [30]. A similar study was conducted on a group of 56 people with HNC who underwent a 7 week RT. The level of miR-181a expression and the values of bioelectric impedance parameters (BIA) were assessed. A significant correlation was found between high miR-181a expression and cancer cachexia in HNC patients. The high level of miR-181a expression along with a low value of the phase angle (PA) was associated with a higher risk of reducing FFM parameters (OR = 4.72; *p* = 0.04) and FFMI (OR = 3.21; *p* = 0.04). Moreover, patients with low PA and high expression of the studied miRNA had shorter OS (HR = 3.18; 95% Cl = 1.19–8.50; *p* = 0.002) [52].

Okugawa et al. analyzed the level of miR-21 in a group of 167 patients with colorectal cancer. RNA extraction and miR-21 expression were performed from serum samples and formalin-fixed, paraffin-embedded tissues. Expression of miR-21 in serum and tissues in colorectal cancer patients with low psoas muscle mass index (PMI) was increased compared with those with high PMI (*p* = 0.031). Moreover, logistic regression analysis demonstrated that serum miR-21 level was a risk factor for decreased PMI in this group (OR = 2.68; *p =* 0.036) [53]. In another study, conducted on 183 patients with colorectal cancer, from all patients, serum specimens and tissue specimens were obtained and the level of miR-203 was analyzed. The serum level of miR-203 was significantly higher in patients with low PMI (*p* = 0.014). Authors suggest that the pre-operative serum expression of this miRNA may be used as a predictive factor of myopenia (OR = 5.16; *p* = 0.002) [54]. Many clinical, genetic or epigenetic factors may be associated with CWL in HNC patients subjected to RT. Patients exposed to higher doses of ionizing radiation (>65Gy) during C-RT are at risk of CWL [55]. Higher risk of CWL >5% was found in patients with BMI <25 (OR: 3.00; *p* < 0.0001), in patients with advanced HNC (tumor stage: T3-T4) (OR = 1.68; *p* = 0.0300) and patients with cancer of the oral cavity, pharyngeal or supraglottic (OR = 3.12; *p* < 0.0001). Higher risk of CWL >10% was also found in younger patients (<60.9 years OR = 0.46; *p* = 0.005) with a BMI >25 (OR = 5.10; *p* < 0.005) and with cancer of the oral cavity, pharyngeal or supraglottic (OR = 3.86; *p* < 0.001) [18]. Mean weight loss during RT was 4.1 (±4.7) kg, which corresponds to 5.4% (±6.1%) of body weight. On average, patients with CWL lost 9.0% (±4.8%) of their body weight. Moreover, patients with CWL had a shorter 5 year OS compared to patients without CWL during RT (HR = 1.3; 95% CI = 1.1–1.6; *p =* 0.01). Patients with CWL had a significantly shorter 5 year disease-specific survival time than those without CWL (HR = 1.7; 95% CI = 1.2–2.3; *p* = 0.001) [16].

Searching for novel biomarkers of nutritional deficiencies may allow for a more efficient diagnosis, timely implementation of nutritional and supportive treatment and the development of new therapies.

As in the case of most studies, our study is not without limitations. Limitations include a small sample size, heterogeneity regarding histopathological diagnosis and treatment before RT (not all patients had surgery), very old patients potentially burdened with sarcopenia were included (can change the clinical picture of cachexia), and lack of data on muscle mass evaluated by densitometry—in terms of laboratory results reflecting inflammation, only CRP was available, the effect of diet or decreased food intake was not assessed (we were not used any nutritional questionnaires), there was a lack of energy expenditure measurements, single miRNA analysis (miRNA profiles may have higher diagnostic value), and the influence of the studied miRNA on their predicted target genes was not evaluated. On the other hand, in our study, we created a more homogenous group by enrolment of only patients undergoing a specific type of RT (IMRT), and only those who received a full dose of treatment.

Taking into consideration that our results do not allow for sufficient explanation of the role of miR-511-3p in the development of nutritional deficiencies in HNC patients and that there are only few papers on this molecule available, further studies on this issue—especially functional—should be warranted. To confirm the predictive value of miR-511-3p, it should be evaluated in a sufficiently large, prospective study group. However, to our knowledge, this is the first study to demonstrate that miR-511-3p may be a biomarker useful in assessing the risk of nutritional deficiencies in patients with HNC undergoing RT.

## 5. Conclusions

Pretreatment miR-511-3p expression may be a biomarker useful in the assessment of the risk of poor nutritional status in patients with HNC undergoing RT.

## Figures and Tables

**Table 1:** General characteristics of the study group.

Variable			Study Group (*n* = 60)
Gender		Male	51 (85.0%)
		Female[M1] [m^2^]	9 (15.0%)
Age [years]		Mean ± standard deviation, median (range)	65 ± 9.26 63 (42–87)
Age [years]	≥ 63		23 (38.3%)
	< 63		37 (61.7%)
Histopathological diagnosis		Squamous cell carcinoma	58 (96.7%)
		Other	2 (3.3%)
Tumor location		Oropharyngeal	23 (38.3%)
		Larynx	33 (55.0%)
		Others	4 (6.7%)
T stage		T1	1 (1.7%)
		T2	8 (13.3%)
		T3	21 (35.0%)
		T4	30 (50.0%)
N stage		N0	18 (30.0%)
		N1	8 (13.3%)
		N2	29 (48.3%)
		N3	5 (8.3%)
M stage		M0	59 (98.3%)
		M1	1 (1.7%)
Disease stage (TNM)		III	16 (26.7%)
		IVA	35 (58.3%)
		IVB	3 (5.0%)
		IVC	6 (10.0%)
Performance status		≤1	51 (85.0%)
		>1	9 (15.0%)
Type of treatment		Surgery + RT	28 (46.7%)
		Surgery + C-RT	18 (30.0%)
		RT alone	8 (13.3%)
		C-RT	6 (10.0%)
Alcohol consumption		Yes	27 (45.0%)
		No	33 (55.0%)
Smoking status		Smoker	44 (73.3%)
		Non-smoker	16 (26.7%)
Smoking status		Current smoker	41 (93.2%)
		Former smoker	3 (6.8%)
Smoking during treatment		Yes	37 (90.2%)
		No	4 (9.8%)
Parenteral nutrition		Yes	7 (11.7%)
		No	53 (88.3%)
Weight [kg]		Mean ± standard deviation, median (range)	65 ± 11.40 66 (43–91)
BMI [kg/m^2^]		Mean ± standard deviation, median (range)	22.94 ± 4.28 22.84 (14.5–34.4)
SGA		A	9 (15.0%)
		B	29 (48.3%)
		C	22 (36.7%)
NRS-2002		2	41 (68.3%)
		3	16 (26.7%)
		4	2 (3.3%)
		5	1 (1.7%)
CWL		Yes	20 (33.3%)
		No	40 (66.7%)
Nutrition risk index (NRI)		Normal	1(1.67%)
		Mild	3 (5.0%)
		Moderate	53 (88.33%)
		Severe	3 (5.0%)
Interval of time between surgery and nutritional assessment [days]		Mean ± standard deviation, median (range)	70.92 ±35.26 63(25–259)
Extent of surgery		Tumor resection	3 (6.52%)
		Tumor resection with lymphadenectomy	43 (93.48%)
Nutritional support in the post-operational period		PEG	2 (3.34%)
		Feeding tube	20 (33.33%)
		No	38 (63.33%)
Nutritional support in the post-operational time [days]		Mean ± standard deviation, median (range)	15.95 ± 4.89 15.50 (9–28)
Oral nutritional support during RT		Yes	3 (5.00%)
		No	57 (95.00%)
Parenteral nutrition during RT		Yes	7 (11.67%)
		No	53 (88.33%)

Abbreviations: BMI—body mass index, C-RT—chemoradiotherapy, CWL—critical weight loss, ECOG—Eastern Cooperative Oncology Group, M—metastatic spread, N—lymph node involvement, NRI—nutrition risk index, NRS-2002—Nutritional Risk Screening 2002, PEG— percutaneous endoscopic gastrostomy, RT—radiotherapy, SGA—subjective global assessment, T—tumor site and size, and TNM—tumor, node, and metastasis staging.

**Table 2:** Comparisons of the relative expression of miR-511-3p depending on nutritional status, evaluation of the diagnostic usefulness of the relative expression of miR-511-3p in predicting the occurrence of nutritional deficiencies and the assessment of the risk of nutritional deficiencies depending on the relative expression of miR-511-3p.

Nutritional Status		Median (Interquartile Range)		ROC Analysis					Risk Analysis			
		Median (Interquartile Range)	*p*	Cut-Off Value	Sensitivity	Specificity	AUC [95% CI]	*p*	Low [<Cut Off] (%)	High [≥Cut Off] (%)	OR [95% CI]	*p*
SGA	A	6.27 (4.35–7.99)	0.0001 *	≤3.12	84.3	88.90	0.90 [0.80–0.96]	<0.0001 *	1 (2.32%)	8 (47.05%)	37.33 [4.14–336.94]	0.0013 *
	B or C	0.93 (0.32–1.91)							42 (97.67%)	9 (52.95%)		
SGA	A or B	1.49 (0.40–5.65)	0.0251 *	≤2.28	100.00	44.70	0.675 [0.54–0.79]	0.0110 *	21 (50.00%)	17 (94.44%)	17.00 [2.07–139.61]	0.0084 *
	C	0.68 (0.32–1.55)							21 (50.00%)	1 (5.56%)		
SGA	B	1.06 (0.31–4.04)	0.3135	≤2.28	100.00	31.03	0.583 [0.44–0.72]	0.3049	20 (48.78%)	9 (90.00%)	9.45 [1.09–81.52]	0.0411 *
	C	0.68 (0.44–1.37)							21 (51.22%)	1 (10.00%)		
NRS-2002	<3	1.06 (0.33–3.55)	0.5945	>0.14	94.70	19.50	0.543 [0.41–0.67]	0.5844	7 (87.50%)	34 (65.38%)	3.71 [0.42–32.52]	0.2371
	>3	1.09 (0.47–3.45)							1 (12.50%)	18 (34.62%)		
CWL	No	1.64 (0.48–5.59)	0.0025 *	≤0.34	50.00	90.00	0.741 [0.61–0.85]	0.0003 *	4 (30.77%)	36 (76.60%)	7.36 [1.89–28.62]	0.0039 *
	Yes	0.51 (0.06–1.22)							9 (69.23%)	11 (23.40%)		

*—statistically significant results. Abbreviations: AUC—area under the curve, CI—confidence interval, CWL—critical weight loss, NRS-2002—Nutritional Risk Screening 2002, OR—odds ratio, ROC—receiver operating characteristic, and SGA— patient-generated subjective global assessment.

**Table 3:** Correlation between demographic, clinical and nutritional variables and the relative expression of miR-511-3p.

Variable	Relative Expression of miR-511-3p	
	rho	*p*
Age [years]	−0.073	0.5768
Weight [kg]	0.114	0.3845
BMI [kg/m^2^]	0.317	0.0136 *
Total protein [g/L]	−0.045	0.7295
Albumin [g/L]	0.322	0.0094 *
Prealbumin[g/dL]	0.281	0.8314
CRP [mg/L]	−0.340	0.0048 *
Transferrin [g/L]	0.160	0.2206
FM [kg]	0.001	0.9946
FM%	0.001	0.9926
FFM [kg]	−0.311	0.0156 *
FFM%	−0.013	0.9208
FFMI [kg/m^2^]	−0.256	0.0486 *
(nFFMI) [kg/m^2^]	−0.232	0.0747
SGA	−0.440	0.0004 *
NRS-2002	0.062	0.6386
T stage	−0.296	0.0218 *
N stage	−0.298	0.0205 *
M stage	−0.064	0.6276
Disease stage (TNM)	−0.443	0.0004 *
NRI	0.317	0.0130 *
Interval of time between surgery and nutritional assessment [days]	−0.001	0.8826
Nutritional support in the post-operational time [days]	−0.250	0.2467

*—statistically significant results. Abbreviations: BMI—body mass index, CRP—C-reactive protein, FFM—fat-free mass, FFMI—fat-free mass index, FM—fat mass, M—metastatic spread, N—lymph node involvement, nFFMI—normalized fat-free mass index, NRI—nutrition risk index, NRS-2002—Nutritional Risk Screening 2002, SGA—subjective global assessment, T—tumor site and size, and TNM—tumor, node, and metastasis staging.

**Table 4:** Influence of demographic, clinical, nutritional and epigenetic variables on overall survival.

Variable	Log-Rank Test			
	Univariable Analysis		Multivariable Analysis ^#^	
	HR [95% CI]	*p*	HR [95% CI]	*p*
Gender (male)	1.61 [0.67–3.85]	0.2931	1.95 [0.59–6.46]	0.2763
Age (≥ 63 years)	1.70 [0.83–3.48]	0.0861	1.62 [0.82–3.22]	0.1695
Smoking history (yes)	0.68 [0.32–1.44]	0.2414	0.74 [0.36–1.51]	0.4061
Smoking during treatment (yes)	0.80 [0.41–1.56]	0.4681	0.80 [0.40–1.58]	0.5219
Alcohol consumption (yes)	0.81 [0.42–1.54]	0.4963	0.73 [0.37–1.43]	0.3587
Performance status (>0)	2.20 [0.79–6.12]	0.0343 *	2.03 [0.87–4.75]	0.1019
Tumor location (oropharyngeal)	1.05 [0.54–2.05]	0.8773	1.07 [0.54–2.11]	0.8431
Tumor location (larynx)	0.91 [0.47–1.73]	0.7497	0.97 [0.49–1.93]	0.9307
T stage (T4)	1.27 [0.66–2.44]	0.4288	1.17 [0.60–2.29]	0.6483
N stage (N1–3)	1.34 [0.68–2.62]	0.3815	1.26 [0.61–2.61]	0.5337
M stage (M1)	15.06 [0.01–27,725.05]	0.0003 *	10.81 [1.04–112.32]	0.0473 *
TNM stage (IV)	1.99 [0.78–5.05]	0.0555	2.08 [0.95–4.55]	0.0666
Parenteral nutrition (yes)	1.88 [0.56–6.28]	0.1654	1.46 [0.51–4.18]	0.4855
Treatment (concurrent C-RT)	1.21 [0.63–2.33]	0.5314	1.26 [0.64–2.45]	0.5013
SGA (C)	1.43 [0.62–3.29]	0.4339	1.45 [0.56–3.74]	0.4473
SGA (BC)	1.00 [0.52–1.93]	0.9961	0.84 [0.41–1.70]	0.6293
NRS-2002 (≥3)	1.23 [0.57–2.64]	0.5451	1.49 [0.68–3.27]	0.3224
CWL (yes)	0.85 [0.42–1.72]	0.6507	0.90 [0.43–1.88]	0.7856
Relative expression of miR-511–3p (high)(≥2.84)	0.93 [0.46–1.86]	0.8225	0.94 [0.45–1.93]	0.8587

#—adjusted for statistically significant variables from univariable analysis. *—statistically significant results. Abbreviations: CI—confidence interval, CWL—critical weight loss, HR—hazard ratio, M—metastatic spread, N—lymph node involvement, NRS-2002—Nutritional Risk Screening, SGA—subjective global assessment, T- T—tumor site and size, and TNM—tumor, node, and metastasis staging.

## Data Availability

Not applicable.

## Supplementary Materials

The following are available online at https://www.mdpi.com/article/10.3390/jcm11030805/s1, Table S1: Association of demographic and clinical variables with the risk of malnutrition according to SGA; Table S2: Association of demographic and clinical variables with the risk of higher nutritional risk according to NRS; Table S3: Association of demographic, clinical and nutritional variables with the risk of CWL; Table S4: Comparison of relative expression of miR-511-3p depending on demographic, clinical and nutritional variables.
