# Supplementary material for: High miR-511-3p Expression as a Potential Predictor of a Poor Nutritional Status in Head and Neck Cancer Patients Subjected to Intensity-Modulated Radiation Therapy
Source: J Clin Med. 2022 Feb 2;11(3):805. doi: 10.3390/jcm11030805 (PMC8836435; doi:10.3390/jcm11030805)
Supplement: Supplementary file 1 [file jcm-11-00805-s001.zip › jcm-1478720-supplementary.pdf]

Table S1. Association of demographic and clinical variables with the risk of malnutrition according to SGA.

Variable		SGA					
		A	B and C	*p* OR [95%CI]	A and B	C	*p* OR [95%CI]
**Gender**	**Male**	6 (11.76%)	45 (88.24%)	0.113	31 (60.78%)	20 (39.22%)	0.3388
	**Female**	3 (33.33%)	6 (66.67%)	3.75 [0.34-19.08]	7 (77.78%)	2 (22.22%)	2.26 [0.42-11.98]
**Age (years)**	**≥63**	4 (17.39%)	19 (82.61%)	0.6832	12 (36.36%)	11 (63.64%)	0.1606
	**<63**	5 (13.51%)	32 (86.49%)	0.74 [0.18-3.11]	26 (70.27%)	11 (29.73%)	2.17 [0.73-6.38]
**Histopathological diagnosis**	**Squamous-cell carcinoma**	8 (13.79%)	50 (86.21%)	0.2108	14 (60.87%)	9 (39.13%)	0.7550
	**Others**	1 (50.00%)	1 (50.00%)	6.25 [0.35-110.29]	24 (64.86%)	13 (35.14%)	1.19 [0.40-3.48]
**Tumor location**	**Oropharyngeal**	6 (26.09%)	17 (73.91%)	0.0707	14 (51.85%)	13 (48.15%)	0.0985
	**Others**	3 (8.11%)	34 (91.89%)	0.25 [0.05-1.12]	24 (72.72%)	9 (27.28%)	2.48 [0.85-7.26]
**Tumor location**	**Larynx**	3 (9.09%)	30 (90.91%)	0.1684	21 (63.63%)	12 (36.36%)	0.9571
	**Others**	6 (22.22%)	21 (77.78%)	2.86 [0.64-12.73]	17 (62.96%)	10 (37.04%)	0.97 [0.34-2.79]
**T stage**	**T4**	-	30 (100.00%)	0.0258*	14 (46.67%)	16 (43.33%)	0.0094*
	**T1-3**	9 (30.00%)	21 (70.00%)	26.95 [1.49-488.36]	24 (80.00%)	6 (20.00%)	4.57 [1.45-14.39]
**N stage**	**N1-3**	6 (14.28%)	36 (85.71%)	0.8131	24 (57.14%)	18 (42.86%)	0.1358
	**N0**	3 (16.67%)	15 (83.33%)	1.20 [0.26-5.44]	14 (77.78%)	4 (22.22%)	2.62 [0.74-9.33]
**M stage**	**M1**	-	1 (100.00%)	0.7321	-	1 (100.00%)	0.3097
	**M0**	9 (15.25%)	50 (84.75%)	0.56 [0.02-14.92]	38 (64.41%)	21 (35.59%)	5.37 [0.21-137.70]
**Disease stage**	**IVA-IVC**	-	6 (100.00%)	0.5085	2 (33.33%)	4 (66.67%)	0.1289
	**III**	9 (16.67%)	45 (83.33%)	2.71 [0.14-52.39]	36 (66.67%)	18 (33.33%)	4.00 [0.67-23.94]
**Performance status**	**>1**	2 (22.22%)	7 (77.78%)	0.5149	4 (44.44%)	5 (55.56%)	0.2117
	**≤1**	7 (13.72%)	44 (86.27%)	0.56 [0.10-3.24]	34 (66.67%)	17 (33.33%)	2.50 [0.59-10.53]
**Alcohol consumption**	**Yes**	3 (11.11%)	24 (88.89%)	0.0449*	14 (51.85%)	13 (48.15%)	0.0985
	**No**	6 (18.18%)	27 (81.82%)	1.78 [0.40-7.90]	24 (72.73%)	9 (27.27%)	2.48 [0.84-7.26]
**Smoking status**	**Smoker**	4 (9.09%)	40 (90.91%)	0.0441*	25 (56.82%)	19 (43.18%)	0.0928
	**Non-smoker**	5 (31.25%)	11 (68.75%)	4.54 [1.04-19.86]	13 (81.25%)	3 (18.75%)	3.29 [0.82-13.22]

* - statistically significant results

Abbreviations: A – well-nourished patients, B – moderately malnourished patients, C – severely malnourished patients, CI - confidence interval, M - metastatic spread, N - lymph node involvement, OR- odds ratio, SGA – subjective global assessment, T - tumor site and size

Table S2. Association of demographic and clinical variables with the risk of higher nutritional risk according to NRS.

Variable		NRS-2002		
		<3	≥3	*p*<br>OR [95%CI]
**Gender**	**Male**	34 (66.67%)	17 (33.33%)	0.5128
	**Female**	7 (77.78%)	2 (22.22%)	1.75 [0.33-9.35]
**Age (years)**	**≥ 63**	16 (73.91%)	7 (26.09%)	0.8715
	**< 63**	25 (67.57%)	12 (32.43%)	0.91 [0.29-2.80]
**Histopathological diagnosis**	**Squamous-cell carcinoma**	40 (69.96%)	18 (31.04%)	0.5799
	**Others**	1 (50.00%)	1 (50.00%)	0.45 [0.02-7.60]
**Tumour location**	**Oropharyngeal**	17 (73.91%)	6 (26.09%)	0.4652
	**Others**	24 (64.86%)	13 (35.14%)	0.65 [0.21-2.06]
**Tumour location**	**Larynx**	21 (63.64%)	12 (36.36%)	0.3890
	**Others**	20 (74.07%)	7 (25.92%)	1.63 [0.53-4.98]
**T stage**	**T4**	21 (70.00%)	9 (30.00%)	0.7814
	**T1-3**	20 (66.67%)	10 (33.33%)	0.86 [0.29-2.55]
**N stage**	**N1-3**	31 (73.81%)	11 (26.19%)	0.1683
	**N0**	10 (55.56%)	8 (44.44%)	0.44 [0.14-1.41]
**M stage**	**M1**	1 (100.00%)	-	0.8243
	**M0**	40 (67.80%)	19 (32.20%)	0.69 [0.02-17.78]
**Disease stage**	**IVA-IVC**	3 (50.00%)	3 (50.00%)	0.3196
	**III**	38 (70.37%)	16 (29.63%)	2.37 [0.43-13.05]
**Performance status**	**>1**	7 (77.78%)	2 (22.22%)	0.5128
	**≤1**	34 (66.67%)	17 (33.33%)	0.57 [0.11-3.05]
**Alcohol consumption**	**Yes**	18 (66.67%)	9 (33.33%)	0.8018
	**No**	23 (69.70%)	10 (30.30%)	1.15 [0.39-3.42]
**Smoking status**	**Smoker**	31 (70.45%)	13 (29.54%)	0.5590
	**Non-smoker**	10 (62.50%)	6 (37.50%)	0.70 [0.21-2.32]

* - statistically significant result

Abbreviations: CI - confidence interval, M - metastatic spread, N - lymph node involvement, NRS-2002 - Nutritional Risk Screening 2002,. OR- odds ratio, T - tumor site and size

Table S3. Association of demographic, clinical and nutritional variables with the risk of CWL.

Variable		CWL		
		No	Yes	*p*<br>OR [95%CI]
Gender	Male	34 (66.67%)	17 (33.33%)	1.0000
	Female	6 (66.67%)	3 (33.33%)	1.00 [0.22-4.50]
Age (years)	≥ 63	16 (69.56%)	7 (30.43%)	0.7075
	< 63	24 (64.86%)	13 (35.14%)	0.81 [0.26-2.46]
Tumour location	Oropharyngeal	9 (39.13%)	14 (60.87%)	0.0007*
	Others	31 (83.78%)	6 (16.22%)	8.04 [2.39-26.97]
Tumour location	Larynx	29 (87.88%)	4 (12.12%)	0.0004*
	Others	11 (40.74%)	16 (59.26%)	0.09 [0.03-0.34]
Histopathological diagnosis	Squamous-cell carcinoma	39 (67.24%)	19 (32.76%)	0.6179
	Others	1 (50.00%)	1 (50.00%)	0.49 [0.03-8.22]
T stage	T4	18 (60.00%)	12 (40.00%)	0.2758
	T1-3	22 (73.33%)	8 (26.67%)	1.83 [0.62-5.45]
N stage	N1-3	27 (64.28%)	15 (35.71%)	0.5512
	N0	13 (72.22%)	5 (27.78%)	1.44 [0.43-4.84]
M stage	M1	1 (100.00%)	-	0.7891
	M0	39 (66.10%)	20 (33.90%)	0.64 [0.02-16.48]
Disease stage	IVA-IVC	5 (83.33%)	1 (16.67%)	0.3777
	III	35 (59.32%)	19 (40.68%)	0.37 [0.04-3.38]
Performance status	>1	6 (66.67%)	3 (33.33%)	1.000
	≤1	34 (66.67%)	17 (33.33%)	1.00 [0.22-4.50]
Alcohol consumption	Yes	18 (66.67%)	9 (33.33%)	1.000
	No	22 (66.67%)	11 (33.33%)	1.00 [0.34-2.94]
Smoking status	Smoker	26 (59.09%)	18 (40.91%)	0.0530
	Non-smoker	14 (87.50%)	2 (12.50%)	4.85 [0.98-23.98]
NRS-2002	≥3	24 (58.54%)	17 (41.46%)	0.0592*
	<3	16 (84.21%)	3 (15.79%)	3.78 [0.95-15.03]
SGA	B and C	34 (66.67%)	17 (33.33%)	1.0000
	A	6 (66.67%)	3 (33.33%)	1.00 [0.22-4.50]
SGA	C	13 (59.09%)	9 (40.91%)	0.3456
	A and B	27 (71.05%)	11 (28.95%)	1.69 [0.56-5.11]
Parenteral nutrition during RT	Yes	5 (71.43%)	2 (28.57%)	0.7766
	No	35 (66.04%)	18 (33.96%)	0.78 [0.14-4.41]

* - statistically significant results

Abbreviations: CI - confidence interval, CWL- critical weight loss, M - metastatic spread, N - lymph node involvement,.NRS-2002- Nutritional Risk Screening 2002, SGA- Subjective Global Assessment, T - tumor site and size, OR- odds ratio, RT - radiotherapy

Table S4. Comparison of relative expression of miR-511-3p depending on demographic, clinical and nutritional variables.

Variable		Relative expression of miR-511-3p Median (interquartile range)	*p*
**Gender**	**Male**	1.01 (0.38-2.91)	0.3569
	**Female**	1.43 (0.56-6.23)	
**Age (years)**	**≥ 63**	1.16 (0.24-4.02)	0.7209
	**< 63**	1.06 (0.43-3.34)	
**Tumour location**	**Oropharyngeal**	0.68 (0.15-3.16)	0.1265
	**Others**	1.29 (0.49-4.04)	
**Tumour location**	**Larynx**	1.36 (0.61-4.05)	0.0946
	**Others**	0.67 (0.15-3.16)	
**Histopathological diagnosis**	**Squamous-cell carcinoma**	1.07 (0.40-3.18)	0.5924
	**Others**	7.34 (-)	
**T stage**	**T1-3**	2.45 (0.68-5.88)	0.0068*
	**T4**	0.68 (0.32-1.56)	
**N stage**	**N0**	0.99 (0.34-2.28)	0.1557
	**N1-3**	1.54 (0.67-6.63)	
**M stage**	**M0**	0.64 (-)	-
	**M1**	1.09 (0.38-3.67)	
**Disease stage**	**III**	1.33 (0.44-4.67)	0.0128*
	**IVA-IVC**	0.32 (0.11-0.49)	
**Performance status**	**≤1**	1.16 (0.44-3.16)	0.6713
	**>1**	0.47 (0.24-8.43)	
**Alcohol consumption**	**Yes**	0.97 (0.44-2.01)	0.3304
	**No**	1.29 (0.36-5.71)	
**Smoking status**	**Smoker**	1.04 (0.33-2.02)	0.1603
	**Non-smoker**	2.70 (0.47-6.14)	
**Extent of surgery**	**Tumor resection**	3.18 (1.43-14.34)	0.1611
	**Tumor resection with lymphadenectomy**	1.16 (0.04-9.80)	
**Nutritional support in the post-operational period**	**No**	0.99 (0.001-14.34)	0.9267
	**Yes[a]**	1.49 (0.04-9.80)	

* - statistically significant results, [a]-PEG or feeding tube .

Abbreviations: M - metastatic spread, N - lymph node involvement, PEG- Percutaneous Endoscopic Gastrostomy, T - tumor site and size.
